# Efficacy and safety of sintilimab combined with histology-specific chemotherapy in advanced NSCLC: a real-world retrospective study

**DOI:** 10.3389/fphar.2026.1811195

**Published:** 2026-06-11

**Authors:** Cong-Ling Chen, Qiong-Qing Chen, Li-Ling Pan, Nan Wang, Jian-Xin Li, Ning-Sheng Liang, Qiong Wu, You-Jia Guo

**Affiliations:** 1 Department of Pharmacy, Guangxi Medical University Cancer Hospital, Nanning, China; 2 Department of Pharmacy, Maternity and Child Health Care of Guangxi Zhuang Autonomous Region, Nanning, China; 3 Guangxi Medical University, Nanning, China; 4 Nanning Nengzhuo Information Technology Service Co., Ltd, Nanning, China

**Keywords:** adenocarcinoma, histological subtype, immunotherapy, non-small cell lung cancer, real-world study, sintilimab, squamous cell carcinoma

## Abstract

**Objectives:**

This study aimed to evaluate whether the clinical efficacy of first-line sintilimab combined with histology-specific chemotherapy (adenocarcinoma [ADC]: pemetrexed/platinum [Pem/Plat]; squamous cell carcinoma [SqCC]: paclitaxel/platinum [Pac/Plat]) differs by histological subtype in advanced non-small cell lung cancer (NSCLC).

**Methods:**

This retrospective, real-world study included 210 patients with advanced NSCLC treated between January 2019 and April 2025 (ADC: n = 124; SqCC: n = 86). The primary endpoints were progression-free survival (PFS) and overall survival (OS). Survival data were analyzed using the Kaplan-Meier method and Cox proportional hazards models, with efficacy heterogeneity tested by incorporating an interaction term.

**Results:**

In the overall population, sintilimab plus chemotherapy significantly improved both PFS (median PFS: 12.30 vs. 7.30 months, HR = 0.547, *P* = 0.001) and OS (median OS: 24.30 vs. 17.93 months, HR = 0.647, *P* = 0.016). Testing for heterogeneity of treatment effect revealed a statistically significant difference in benefit between histological subtypes (PFS interaction *P* = 0.025; OS interaction *P* = 0.001). In patients with SqCC, the combination conferred significant PFS (median PFS: 17.17 vs. 6.10 months, HR = 0.382, *P* = 0.001) and OS benefits (median OS: 52.33 vs. 11.57 months, HR = 0.312, *P* < 0.001) and emerged as the strongest independent favorable prognostic factor in multivariable analysis. However, in patients with ADC, the combination only showed a trend toward improved PFS (median PFS: 11.53 vs. 8.23 months, HR = 0.641, *P* = 0.057) and did not lead to a significant OS benefit (median OS: 21.87 vs. 30.50 months, HR = 1.043, *P* = 0.865). The presence of ≥2 distant metastatic sites was an independent negative prognostic factor in ADC. The safety profile was consistent with the known spectrum of the agents, with no new safety signals identified.

**Conclusion:**

In real-world practice, the survival benefit of sintilimab combined with histology-specific chemotherapy is significantly dependent on the histological subtype in advanced NSCLC. Patients with SqCC derived a clear and substantial survival advantage, whereas the benefit for patients with ADC was more limited and influenced by high metastatic burden. These findings suggest that in the immunotherapy era, histological subtyping remains a crucial yet insufficient guide for clinical decision-making, and future strategies should integrate subtype-specific biomarkers for further treatment optimization.

## Introduction

Lung cancer remains the leading cause of cancer-related mortality worldwide, with non-small cell lung cancer (NSCLC) accounting for approximately 85% of all cases (Sung et al., 2021). The majority of patients are diagnosed at an advanced or metastatic stage, for whom systemic therapy represents the cornerstone of treatment (Riely et al., 2024; Chen et al., 2024). The advent of immune checkpoint inhibitors (ICIs), particularly those targeting the programmed death-1 (PD-1)/programmed death-ligand 1 (PD-L1) axis, has revolutionized the therapeutic landscape for advanced NSCLC (Gandhi et al., 2018; Reck et al., 2016).

Sintilimab is a highly selective humanized IgG4 anti-PD-1 monoclonal antibody approved by China’s National Medical Products Administration (Guo et al., 2025). It yields robust efficacy and a manageable safety profile as first-line chemotherapy combination therapy for advanced NSCLC (Wang et al., 2019). Based on the landmark ORIENT-11 and ORIENT-12 trials, sintilimab plus pemetrexed/platinum (Pem/Plat) and paclitaxel/platinum (Pac/Plat) have become standard first-line regimens for advanced adenocarcinoma (ADC) and squamous cell carcinoma (SqCC), respectively (Zhou et al., 2021; Yang et al., 2020). This therapeutic strategy is strictly histology-driven. Given the inherent differences in chemosensitivity among distinct NSCLC subtypes, the principle of selecting chemotherapy backbones according to histological type has been well established even prior to the immunotherapy era (Rizvi et al., 2018; Scagliotti et al., 2008).

However, within this histology-guided framework, a critical and unresolved question remains: does the intrinsic efficacy of the PD-1 inhibitor sintilimab differ when combined with its distinct, histologically matched chemotherapy backbones? Although the overall survival (OS) benefit of adding sintilimab to chemotherapy has been established in the respective trial populations, a systematic investigation into potential nuanced differences in treatment benefit between patients with ADC and those with SqCC is lacking (Zhou et al., 2021; Yang et al., 2020). Emerging evidence suggests that immunotherapy efficacy may not be uniform across NSCLC subtypes, likely influenced by histology-specific variations in the tumor immune microenvironment, mutational landscape, and oncogenic pathways (Rizvi et al., 2018). Furthermore, real-world data comparing the safety profiles of sintilimab-based regimens between ADC and SqCC remain scarce.

Therefore, this retrospective real-world study explored, for the first time, the differences in efficacy and safety of sintilimab combined with histology-specific chemotherapy between ADC and SqCC subtypes in advanced NSCLC. We further analyzed potential factors contributing to the observed efficacy disparities and performed a histology-stratified safety assessment, aiming to provide evidence for individualizing treatment responses and refining adverse event management.

## Methods

### Patients

The present study was a retrospective study conducted at the Guangxi Medical University Affiliated Cancer Hospital. The medical records of consecutive patients who initiated therapy between January 2019 and April 2025 were reviewed. Records for patients with NSCLC (according to the 2015 World Health Organization classification), incurable stage IIIB-IV tumors (according to the 8th edition AJCC/IASLC classification) (Goldstraw et al., 2016), and at least one measurable lesion according to the Response Evaluation Criteria in Solid Tumors (RECIST), version 1.1 (Eisenhauer et al., 2009). Included patients received at least 2 cycles of sintilimab combined with platinum-based doublet chemotherapy or platinum-based doublet chemotherapy alone. Platinum-based doublet regimens included Pem/Plat (pemetrexed combined with carboplatin or cisplatin) and Pac/Plat (paclitaxel combined with carboplatin or cisplatin). Patients were excluded if they had: (1) other primary tumor types; (2) severe comorbidities involving vital organs (heart, brain, etc.); (3) long-term use of glucocorticoids or immunosuppressants; or (4) immunodeficiency disorders. The study was reviewed and approved by the ethics committee of Guangxi Medical University Affiliated Cancer Hospital (KY2023846). The ethics committee at each institution approved this study. The study protocol was conducted according to the principles of the Declaration of Helsinki.

### Assessments

Patient data were retrospectively extracted from electronic medical records and included demographics, clinical characteristics, treatment regimens, survival outcomes, and adverse events (AEs). The Eastern Cooperative Oncology Group performance status (ECOG PS) was assessed at baseline, with tumor imaging performed every 2–3 treatment cycles for response evaluation. Objective response rate (ORR) is defined as the proportion of patients achieving complete response (CR) or partial response (PR), and the disease control rate (DCR) as the proportion with CR, PR, or stable disease (SD). The co-primary endpoints of this study were progression-free survival (PFS) and overall survival (OS). PFS was defined as the time from treatment initiation to disease progression, death from any cause, or the data cutoff date (whichever occurred first). OS was defined as the time from treatment initiation to death from any cause or the data cutoff date of April 30, 2025. AEs were graded according to the Common Terminology Criteria for Adverse Events (CTCAE), version 5.0.

### Statistical analysis

Statistical analyses were performed using SPSS software (version 26.0). Continuous variables are expressed as mean ± standard deviation and were compared using the independent samples t-test. Categorical variables are expressed as frequency (percentage) and were compared using the chi-square test or Fisher’s exact test, as appropriate. Survival curves were plotted using the Kaplan-Meier method, and differences between groups were assessed with the log-rank test. Hazard ratios (HRs) and 95% confidence intervals (CIs) were calculated using the Cox proportional hazards regression model. To test the prespecified hypothesis of treatment efficacy heterogeneity, an interaction term between the treatment regimen and histological subtype was incorporated into the Cox models. Subsequently, multivariable Cox analyses were performed separately for the adenocarcinoma and squamous cell carcinoma subgroups, adjusting for key clinical covariates. All tests were two-tailed, and a *P* value of less than 0.05 was considered statistically significant.

## Results

### Baseline characteristics

Baseline characteristics of the overall study population and histological subgroups are summarized in Tables 1, 2, respectively. Among the 210 enrolled patients, 115 received sintilimab plus chemotherapy and 95 received chemotherapy alone. Characteristics including age, sex, smoking history, and specific metastatic sites were comparable between groups (all *P* > 0.05). However, the sintilimab-combination group had significantly higher proportions of patients with ECOG PS 1 (88.7% vs. 11.3%, *P* = 0.034) and stage IV disease (86.1% vs. 13.9%, *P* < 0.001), but lower rates of driver gene positivity (35.7% vs. 58.9%, *P* = 0.001) and PD-L1 positivity (60.0% vs. 84.2%, *P* < 0.001). In the ADC cohort (n = 124), the treatment groups were balanced for most features except for stage IV disease (94.4% vs. 76.9%, *P* = 0.006) and PD-L1 positivity (54.2% vs. 76.9%, *P* = 0.009). In the SqCC cohort (n = 86), the groups differed in ECOG PS 0 (4.7% vs. 25.6%, *P* = 0.014), stage IIIB/C disease (27.9% vs. 48.8%, *P* = 0.046), and PD-L1 positivity (69.8% vs. 93.0%, *P* = 0.006). These imbalanced prognostic factors were adjusted for in subsequent multivariable analyses.

**TABLE 1:** Baseline characteristics of the overall study population.

Characteristics	Sintilimab + chemotherapy (N = 115)	Chemotherapy (N = 95)	*P* value
Age (≥60), n (%)	60 (52.2)	52 (54.7)	0.711
Sex (male/female)	91/24	74/21	0.828
Smoking History, n (%)	69 (60.0)	57 (60.0)	1.000
ECOG PS, n (%)	​	​	0.034
0	74 (77.9)	21 (22.1)	​
1	102 (88.7)	13 (11.3)	​
Disease stage, n (%)	​	​	<0.001
IIIB/C	16 (13.9)	33 (34.7)	​
IV	99 (86.1)	16 (13.9)	​
Metastatic Sites, n (%)			
Brain	14 (12.2)	11 (11.6)	0.895
Liver	20 (17.4)	13 (13.7)	0.463
Bone	33 (28.7)	29 (30.5)	0.772
Adrenal gland	17 (14.8)	10 (10.5)	0.359
≥2 distant sites	49 (42.6)	32 (33.7)	0.186
Driver gene positive, n (%)	41 (35.7)	56 (58.9)	0.001
PD-L1 positive, n (%)	69 (60.0)	80 (84.2)	<0.001
Radiotherapy History	15 (13.0)	14 (14.7)	0.723
Lung Cancer surgery History, n (%)	12 (10.4)	9 (9.5)	0.817

**TABLE 2:** Baseline characteristics stratified by histological subtype.

Characteristics	Sintilimab + pem/Plat (N = 72)	Pem/Plat alone (N = 52)	*P* value	Sintilimab + Pac/Plat (N = 43)	Pac/Plat alone (N = 43)	*P* value
Age (≥60), n (%)	34 (47.2)	28 (53.8)	0.467	21 (48.8)	15 (34.9)	0.190
Sex (male/female)	57/15	35/17	0.136	34/9	39/4	0.132
Smoking History, n (%)	39 (54.2)	23 (44.2)	0.275	30 (69.8)	34 (79.1)	0.323
ECOG PS, n (%)	​	​	0.563	​	​	0.014
0	11 (15.3)	10 (19.2)	​	2 (4.7)	11 (25.6)	​
1	61 (84.7)	42 (80.8)	​	41 (95.4)	32 (74.4)	​
Disease stage, n (%)	​	​	0.006	​	​	0.046
IIIB/C	4 (5.6)	12 (23.1)	​	12 (27.9)	21 (48.8)	​
IV	68 (94.4)	40 (76.9)	​	31 (72.1)	22 (51.2)	​
Metastatic Sites, n (%)						
Brain	12 (16.7)	10 (19.2)	0.712	2 (4.7)	1 (2.3)	0.557
Liver	11 (15.3)	8 (15.4)	0.987	9 (20.9)	5 (11.6)	0.243
Bone	24 (33.3)	15 (28.8)	0.595	9 (20.9)	14 (32.6)	0.223
Adrenal gland	14 (19.4)	7 (13.5)	0.381	3 (7.3)	3 (7.0)	1.000
≥2 distant sites	34 (47.2)	20 (38.5)	0.332	15 (34.9)	12 (27.9)	0.486
Driver gene positive, n (%)	17 (23.6)	10 (19.2)	0.702	21 (48.8)	34 (79.1)	0.004
PD-L1 positive, n (%)	39 (54.2)	40 (76.9)	0.009	30 (69.8)	40 (93.0)	0.006
Radiotherapy History	11 (15.3)	6 (11.5)	0.550	4 (9.3)	8 (18.6)	0.213
Lung Cancer surgery History, n (%)	8 (11.1)	8 (15.4)	0.484	4 (9.3)	1 (2.3)	0.167

ECOG PS, Eastern Cooperative Oncology Group performance status; PD-L1, programmed death-ligand 1.

### Survival outcomes

The median follow-up time was 20.85 months (2.93–79.13 months). In the overall population, sintilimab plus chemotherapy significantly improved both ORR (33.0% vs. 9.4%, *P* < 0.001) and DCR (95.7% vs. 88.4%, *P* = 0.045) compared to chemotherapy alone (Table 3). This benefit was consistently observed in both histological subtypes. For ADC, the combination therapy significantly improved ORR (31.9% [95% CI, 21.5–44.0] vs. 13.5% [5.6–25.8]; *P* = 0.013) and DCR (97.2% [90.3–99.7] vs. 88.5% [76.6–95.6]; *P* = 0.049). In the SqCC cohort, the improvement in ORR was particularly pronounced (34.9% [21.0–50.9] vs. 4.7% [0.6–15.8]; *P* = 0.001), while a high DCR was maintained in both treatment groups (93.0% [80.9–98.5] vs. 88.4% [74.9–96.1]; *P* = 0.474) (Table 4).

**TABLE 3:** Treatment response of the overall study population.

Variable	Sintilimab + chemotherapy (N = 115)	Chemotherapy (N = 95)	*P* value
Best overall response, n (%)			
Complete response	0 (0.0)	0 (0.0)	​
Partial response	38 (33.0)	9 (9.4)	​
Stable disease	72 (62.6)	75 (78.9)	​
Progressive disease	5 (4.3)	11 (11.6)	​
Objective response rate, n (%) [95% CI]	38 (33.0) [24.5–42.7]	9 (9.4) [4.2–17.5]	<0.001
Disease control rate, n (%) [95% CI]	110 (95.7) [90.9–98.5]	84 (88.4) [80.2–93.9]	0.045

**TABLE 4:** Treatment response stratified by histological subtype.

Variable	Sintilimab + pem/Plat (N = 72)	Pem/Plat alone (N = 52)	*P* value	Sintilimab + pac/Plat (N = 43)	Pac/Plat alone (N = 43)	*P* value
Best overall response, n (%)						
Complete response	0 (0.0)	0 (0.0)	​	0 (0.0)	0 (0.0)	​
Partial response	23 (31.9)	7 (13.5)	​	15 (34.9)	2 (4.7)	​
Stable disease	47 (65.3)	39 (75.0)	​	25 (58.1)	36 (83.7)	​
Progressive disease	2 (2.8)	6 (11.5)	​	3 (7.0)	5 (11.6)	​
Objective response rate, n (%) [95% CI]	23 (31.9) [21.5–44.0]	7 (13.5) [5.6–25.8]	0.013	15 (34.9) [21.0–50.9]	2 (4.7) [0.6–15.8]	0.001
Disease control rate, n (%) [95% CI]	70 (97.2) [90.3–99.7]	46 (88.5) [76.6–95.6]	0.049	40 (93.0) [80.9–98.5]	38 (88.4) [74.9–96.1]	0.474

CI, confidence interval.

**FIGURE 1:**
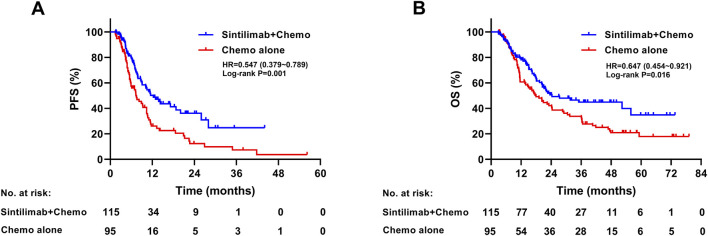
Kaplan-Meier estimates of survival in the overall population. **(A)** Progression-free survival (PFS). **(B)** Overall survival (OS). Chemo, Chemotherapy; PFS, Progression-Free Survival; OS, Overall Survival; CI, confidence interval; HR, hazard ratio.

**FIGURE 2:**
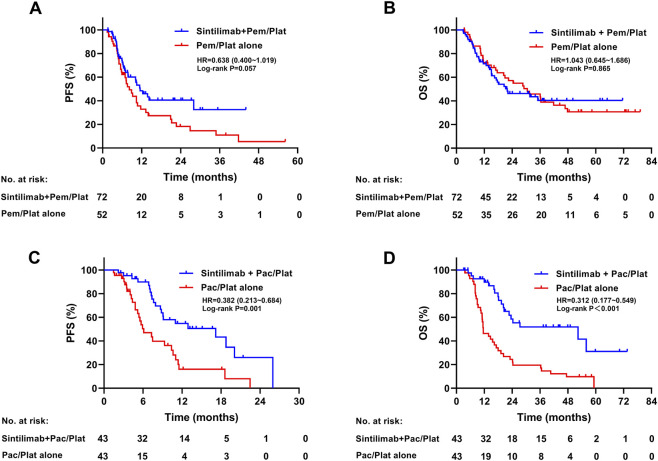
Kaplan-Meier estimates of survival stratified by histological subtype. **(A)** PFS in patients with ADC. **(B)** OS in patients with ADC. **(C)** PFS in patients with SqCC. **(D)** OS in patients with SqCC. PFS, Progression-Free Survival; OS, Overall Survival; CI, confidence interval; HR, hazard ratio.

In the overall population, Kaplan-Meier analysis demonstrated that sintilimab plus chemotherapy significantly improved both PFS (Figure 1A) and OS (Figure 1B) compared with chemotherapy alone (median PFS: 12.30 vs. 7.30 months, HR = 0.547, 95% CI 0.379–0.789, *P* = 0.001; median OS: 24.30 vs. 17.93 months, HR = 0.647, 95% CI 0.454–0.921, *P* = 0.016). In ADC, the sintilimab plus Pem/Plat group exhibited a non-significant trend toward improved PFS (unadjusted HR = 0.636, *P* = 0.057) (Figure 2A) and no OS benefit (unadjusted HR = 1.043, *P* = 0.865) (Figure 2B). In SqCC, the sintilimab plus Pac/Plat group achieved significantly prolonged PFS (unadjusted HR = 0.382, log-rank *P* = 0.001) (Figure 2C) and OS (unadjusted HR = 0.312, *P* < 0.001) relative to the Pac/Plat alone group (Figure 2D).

### Heterogeneity analysis and multivariable analysis

Given the apparent differential outcomes observed between ADC and SqCC subgroups, we formally tested for heterogeneity in treatment effect by histology using a multivariable Cox regression model incorporating an interaction term. A statistically significant interaction between the treatment regimen and histological subtype was confirmed for both PFS (interaction HR = 0.413, 95% CI 0.191–0.894, *P* = 0.025) and OS (interaction HR = 0.270, 95% CI 0.125–0.583, *P* = 0.001) (Table 5). This indicates that the magnitude of survival benefit from adding sintilimab to chemotherapy was significantly dependent on histology.

**TABLE 5:** Multivariable Cox regression analysis with interaction testing for PFS and OS.

Characteristic	​	PFS	​	​	OS	
​	HR	95% CI	P value	HR	95% CI	P vlaue
Treatment	0.618	0.377–1.014	0.057	0.964	0.584–1.590	0.884
Histology	1.864	1.073–3.241	0.027	2.117	1.227–3.652	0.007
Treatment* Histology	0.413	0.191–0.894	0.025	0.270	0.125–0.583	0.001
Age	0.755	0.498–1.145	0.186	0.825	0.547–1.244	0.359
Sex	1.370	0.771–2.434	0.283	1.382	0.778–2.453	0.270
ECOG PS	1.473	0.874–2.483	0.146	1.573	0.926–2.673	0.094
Smoking History	0.813	0.474–1.395	0.452	1.398	0.863–2.267	0.174
Disease Stage	1.510	0.883–2.582	0.132	1.603	1.003–2.562	0.049
≥2 Distant Sites	3.995	2.617–6.098	<0.001	1.742	1.170–2.594	0.006
Driver Gene	1.576	1.034–2.403	0.035	1.396	0.928–2.100	0.109
PD-L1	1.336	0.835–2.140	0.227	1.190	0.760–1.864	0.447

ECOG PS, Eastern Cooperative Oncology Group performance status; PD-L1, programmed death-ligand 1; CI, confidence interval.

Treatment*Histology = Interaction between treatment regimen and histological subtype.

**FIGURE 3:**
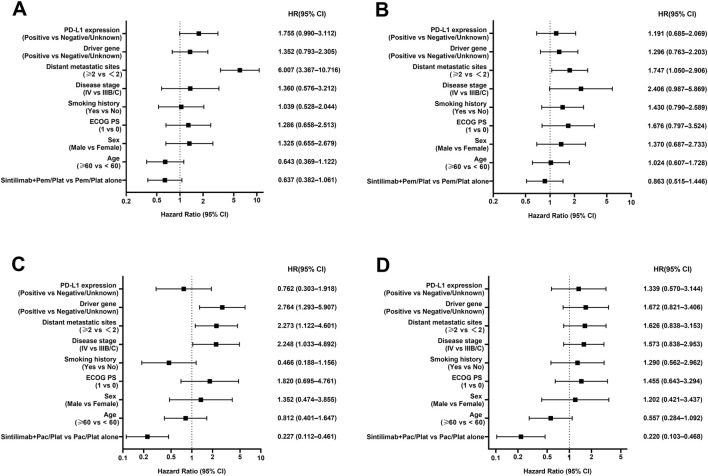
Forest plots of multivariable Cox regression analyses within histological subgroups. **(A)** Factors associated with PFS in ADC. **(B)** Factors associated with OS in ADC. **(C)** Factors associated with PFS in SqCC. **(D)** Factors associated with OS in SqCC. ECOG PS, Eastern Cooperative Oncology Group performance status; PD-L1, programmed death-ligand 1; CI, confidence interval.

In ADC, the sintilimab plus Pem/Plat group exhibited a non-significant trend toward improved PFS (unadjusted HR = 0.636, *P* = 0.057) (Figure 2A) and no OS benefit (unadjusted HR = 1.043, *P* = 0.865) (Figure 2B). On multivariate analysis, the presence of two or more distant metastatic sites (≥2 distant sites) was independently associated with worse survival in this subtype (adjusted HR for PFS = 6.007, for OS = 1.747) (Figures 3A, B). These results collectively demonstrate a differential therapeutic response and distinct prognostic architecture based on histology.

In SqCC, the sintilimab plus Pac/Plat group achieved significantly prolonged PFS (unadjusted HR = 0.382, log-rank *P* = 0.001) (Figure 2C) and OS (unadjusted HR = 0.312, *P* < 0.001) relative to the Pac/Plat alone group (Figure 2D). This treatment benefit remained the strongest independent prognostic factor after multivariate adjustment for both PFS (adjusted HR = 0.227) and OS (adjusted HR = 0.220), with additional risk factors for poorer PFS including advanced disease stage (adjusted HR = 2.248), ≥2 distant sites (adjusted HR = 2.273), and positive driver gene status (adjusted HR = 2.764) (Figures 3C, D).

### Safety

In the ADC cohort, the incidence of any-grade AEs was significantly higher in the sintilimab plus Pem/Plat group than in the Pem/Plat alone group (76.39% vs. 55.77%, *P* = 0.015) (Table 6). However, the rates of grade 3–4 AEs were comparable between the two groups (18.06% vs. 17.31%, *P* = 0.914). Any-grade immune-related adverse events (irAEs) occurred exclusively in the sintilimab plus Pem/Plat group (19.44% vs. 0%, *P* = 0.001), with immune-mediated pneumonitis being the most notable (9.62%), and all cases were grade 3–4. Anemia was the most common hematologic toxicity overall, with a numerically higher incidence in the sintilimab plus Pem/Plat group (67.31% vs. 34.62%, *P* = 0.120). Rates of leukopenia, neutropenia, and thrombocytopenia were similar between groups. For non-hematologic toxicities, nausea/vomiting was more frequent in the Pem/Plat alone group (23.08% vs. 13.46%, *P* = 0.042), while elevated transaminases occurred more often in the sintilimab plus Pem/Plat group (34.62% vs. 13.46%, *P* = 0.114).

**TABLE 6:** Adverse events in the ADC cohort.

Adverse events category	Sintilimab + pem/Plat (N = 72)		Incidence (%)	Pem/Plat alone (N = 52)		Incidence (%)	*P* Value
	Cases (1–2 Grade)	Cases (3–4 Grade)		Cases (1–2 Grade)	Cases (3–4 Grade)		
Any-grade AEsb	55		76.39	29		55.77	0.015
Grade 3–4 AEs	13		18.06	9		17.31	0.914
Any-grade irAEs	14		19.44	0		0	0.001
Hematological toxicity							
Anemia	33	2	67.31	15	3	34.62	0.120
Leukopenia	21	1	42.31	11	2	25.00	0.498
Neutropenia	11	3	26.92	8	3	21.15	0.815
Thrombocytopenia	3	2	9.62	0	2	3.85	0.731
Non-hematological toxicity							
Transaminase elevation	16	2	34.62	5	2	13.46	0.114
Nausea and vomiting	7	0	13.46	3	9	23.08	0.042
Fever	1	1	3.85	2	0	3.85	1.000
Diarrhea	3	1	7.69	0	0	0	0.139
Myocardial enzyme elevation	4	0	7.69	1	0	1.92	0.581
Electrocardiogram changes	3	0	5.77	1	0	1.92	0.855
irAEs							
Immune-related pneumonia	0	5	9.62	0	0	0	0.074
Immune-related cutaneous toxicity	3	1	7.69	0	0	0	0.139
Immune-related myocarditis	2	0	3.85	0	0	0	0.509
Hypothyroidism	4	0	7.69	0	0	0	0.139
Adrenal cortical insufficiency	1	0	1.92	0	0	0	1.000
Immune-related liver injury	1	0	1.92	0	0	0	1.000

AEs, Adverse Events; irAEs, Immune-related Adverse Events.

In the SqCC cohort, the incidence of any-grade AEs was numerically higher in the Pac/Plat alone group than in the sintilimab plus Pac/Plat group (86.05% vs. 72.09%, *P* = 0.112), with comparable rates of grade 3–4 events (13.95% vs. 11.63%, *P* = 0.747) (Table 7). IrAEs were reported only in the sintilimab plus Pac/Plat group (16.28% vs. 0%, *P* = 0.012). Hypothyroidism was the most frequent irAE (11.63%), followed by pneumonitis (6.98%). All irAEs in this cohort were grade 1–2. Anemia was again the most common hematologic event, with no significant difference between groups (30.23% vs. 34.88%, *P* = 0.645). Rates of leukopenia, neutropenia, and thrombocytopenia were numerically higher in the Pac/Plat alone group, though differences were not statistically significant. Elevated transaminases occurred more frequently in the sintilimab plus Pac/Plat group (23.26% vs. 11.63%, *P* = 0.155), while other non-hematologic toxicities, including peripheral neuropathy and rash, were similar between groups.

**TABLE 7:** Adverse events in the SqCC cohort.

Adverse events category	Sintilimab + pac/Plat (N = 43)		Incidence (%)	Pac/Plat alone (N = 43)		Incidence (%)	P Value
	Cases (1–2 Grade)	Cases (3–4 Grade)		Cases (1–2 Grade)	Cases (3–4 Grade)		
Any-grade AEs	31	72.09	​	37		86.05	0.112
Grade 3–4 AEs	5	11.63	​	6		13.95	0.747
Any-grade irAEs	7	16.28	​	0		0	0.012
Hematological toxicity							
Anemia	15	0	34.88	12	1	30.23	0.645
Leukopenia	4	1	11.63	7	5	27.91	0.058
Neutropenia	2	3	11.63	6	4	23.26	0.155
Thrombocytopenia	3	1	9.30	5	3	18.61	0.315
Non-hematological toxicity							
Transaminase elevation	9	1	23.26	3	2	11.63	0.155
Nausea and vomiting	1	0	2.33	2	0	4.65	1.000
Peripheral neurotoxicity	4	1	11.62	4	2	13.95	0.241
Rash	4	0	9.30	1	0	2.33	1.000
irAEs							
Immune-related pneumonia	3	0	6.98	0	0	0	0.055
Hypothyroidism	5	0	11.63	0	0	0	1.000
Immune-related liver injury	1	0	2.32	0	0	0	0.747
Immune-related renal injury	1	0	2.32	0	0	0	0.357
Immune-related cutaneous toxicity	1	0	2.32	0	0	0	1.000

AEs, Adverse Events; irAEs, Immune-related Adverse Events.

## Discussion

This retrospective real-world study evaluated the efficacy and safety of first-line sintilimab combined with histology-specific chemotherapy in advanced NSCLC. In the overall population, the combination regimen significantly improved both PFS (median: 12.30 vs. 7.30 months; HR = 0.547) and OS (median: 24.30 vs. 17.93 months; HR = 0.647), validating the real-world effectiveness of this strategy when translated from strict trial settings into routine clinical practice. However, deeper analysis by histological subgroup revealed nuanced differences and important extensions compared with clinical trial data. In the SqCC subgroup, the survival benefit observed in this study (median OS: 52.33 vs. 11.57 months; HR = 0.312) was numerically greater than that reported in the pivotal ORIENT-12 trial (HR ≈ 0.57), suggesting potentially enhanced efficacy in a broader, more heterogeneous real-world population (Zhou et al., 2021). Conversely, in the ADC subgroup, although the combination therapy improved the objective response rate (31.9% vs. 13.5%) and showed a trend toward better PFS (HR = 0.641, P = 0.057), it did not translate into a significant OS benefit (HR = 1.043, P = 0.865). This finding differs from the clear OS advantage demonstrated in ORIENT-11 (median OS: 24.2 vs. 16.8 months; HR = 0.65). (Yang et al., 2020). These similarities and differences highlight the unique value of real-world evidence: it can both verify the general conclusions of clinical trials and uncover efficacy variations and patient subpopulations that may not be fully observed in controlled trial settings, thereby reflecting the complexity of actual clinical practice (Yan et al., 2025).

Several real-world studies have reported on sintilimab plus chemotherapy. A multicenter study reported a median OS of 26.9 months and a median PFS of 8.4 months for first-line sintilimab plus chemotherapy (Nagasaka et al., 2022). Another study reported a median PFS of 9.8 months for sintilimab in first-line SqCC treatment (Lin et al., 2023). However, most of these studies performed a single comparison (combination therapy vs. chemotherapy alone) without directly comparing the incremental benefit between ADC and SqCC. Our study has four distinguishing features that, to our knowledge, have not been systematically addressed before. First, by providing a head-to-head comparison between the two histological subtypes, we found that SqCC patients derived a substantial survival advantage (OS HR = 0.312), whereas ADC patients showed limited OS benefit (HR = 1.043), which was further constrained by high metastatic burden. Second, by incorporating a treatment-by-histology interaction term into the Cox regression model, we quantitatively demonstrate that the survival benefit of sintilimab-based immunochemotherapy is significantly dependent on histological subtype (P for interaction: PFS = 0.025, OS = 0.001). This finding offers a novel evidence base for individualized treatment decisions. Third, unlike strictly controlled randomized trials, our study captures genuine clinical complexity. We observed a “reverse selection bias,” in which patients with more favorable baseline characteristics (e.g., better ECOG PS, earlier clinical stage, higher PD-L1 positivity) were paradoxically more likely to receive chemotherapy alone (Aguiar et al., 2017; Resuli et al., 2025; Wenfan et al., 2023). Additionally, some driver-gene-positive patients did not receive first-line targeted therapy due to delays in genomic testing, reimbursement restrictions, or clinical preference for rapid tumor control. These observations reflect the realities of clinical decision-making in China. Fourth, this is the first study to compare adverse event profiles between ADC and SqCC in patients receiving sintilimab plus histology-specific chemotherapy. We found that immune-mediated pneumonitis occurred more frequently and with higher severity (all grade 3–4) in the ADC cohort (9.62%), whereas SqCC patients experienced predominantly mild (grade 1–2) hypothyroidism (11.63%). These histology-specific safety patterns provide practical guidance for adverse event monitoring and management.

The observed efficacy heterogeneity can be attributed to multiple factors. Differences in tumor biological characteristics contribute to this discrepancy. SqCC typically harbors a higher tumor mutational burden and a more active “immune-hot” microenvironment, which may render it more sensitive to immunotherapy combinations (Feng et al., 2025; Rizvi et al., 2015; Wang et al., 2025). In contrast, this study identified the presence of≥2 distant sites as an exceptionally strong independent poor prognostic factor in ADC (adjusted HR for PFS = 6.007). The impact of this factor may partially explain the lack of a significant OS benefit in this subgroup, as a high metastatic burden could overwhelm any potential gain from immunotherapy.

The safety profile observed in this study was overall consistent with the known safety spectrum of sintilimab. A recent meta-analysis indicated that compared with chemotherapy alone, sintilimab combination therapy significantly increased the risk of hypothyroidism, pneumonitis, and rash (with risk increases of 3.70-fold, 2.22-fold, and 1.58-fold, respectively), while showing no significant difference in hematologic toxicity or liver injury (Pan et al., 2025). Our findings align with this overall trend. Notably, immune-mediated pneumonitis emerged as the most prominent immune-related adverse event in the ADC cohort (9.62%, all grade 3–4), compared with a lower incidence and severity in the SqCC cohort (6.98%, all grade 1–2). This observation echoes real-world reports such as that by Li et al. and suggests that both histological subtype and the specific concomitant chemotherapy agent (e.g., pemetrexed) may influence the risk of particular toxicities (Li et al., 2024). Nevertheless, the incidence of severe adverse events was generally manageable, and no new safety signals were identified.

Several limitations of this retrospective analysis should be acknowledged. First, histological subtype is inherently matched with distinct chemotherapy regimens following clinical guidelines, making it impossible to distinguish their independent effects. Interaction analysis revealed significant differences in the incremental benefit of sintilimab across subgroups (*P* = 0.001 for OS), and all conclusions are confined to the overall performance of full histology-tailored treatment regimens. Second, the retrospective design carries an inherent risk of selection bias and unmeasured confounding. Third, although the sample size was substantial, it may still limit the statistical power for certain subgroup analyses. Fourth, the lack of comprehensive biomarker data (e.g., detailed PD-L1 expression levels, TMB) for all patients precluded a more granular predictive analysis within each subtype. Finally, the single-center nature of the study may affect the generalizability of the findings, warranting validation in larger, multi-center real-world cohorts.

In conclusion, this real-world study demonstrates that the survival benefit of first-line sintilimab combined with histology-specific chemotherapy is significantly dependent on the histological subtype in advanced NSCLC. Patients with SqCC derived a pronounced and independent survival advantage, whereas the benefit for patients with ADC was more limited and significantly influenced by high metastatic burden. These findings reinforce that histology remains a crucial, yet insufficient, guide in the immunotherapy era and underscore the imperative for continued research into subtype-specific predictive biomarkers to refine and personalize treatment strategies for advanced NSCLC.

## Conclusion

This real-world study demonstrates a significant dependence of survival benefit on histological subtype for first-line sintilimab combined with histology-specific chemotherapy in advanced NSCLC. Patients with squamous cell carcinoma (SqCC) derived a pronounced and independent survival advantage, strongly supporting this regimen as a standard first-line option. In contrast, the benefit for patients with adenocarcinoma (ADC) was more limited and significantly influenced by high metastatic burden. These findings underscore that histological subtyping remains a crucial, yet insufficient, guide in the immunotherapy era. Future strategies should therefore integrate more refined, subtype-specific biomarkers to enable truly personalized therapy for advanced NSCLC.

## Data Availability

The raw data supporting the conclusions of this article will be made available by the authors, without undue reservation.
